# Simultaneous occurrence of five prothrombotic induced vaso-occlusive phenomena and focal nodular hyperplasia due to prolonged use of combined oral contraceptive pills

**DOI:** 10.1259/bjrcr.20170070

**Published:** 2017-12-15

**Authors:** Martin Ian Kamanda, Muriithi Ian Mathenge

**Affiliations:** M.P Shah Hospital, University of Nairobi, Nairobi, Kenya

## Abstract

This is a rare case of a patient simultaneously presenting with five complications associated with the prolonged (5 years) use of combined oral contraceptives. The two main ingredients of combined oral contraceptives responsible for thromboembolism are estrogen (estradiol) and progestogen (progestin). Progestins are linked with occlusion of arteries while estrogens can induce both arterial and venous thrombosis. This case represents a patient with portal vein thrombosis, superior mesenteric vein thrombosis, coeliac artery occlusion, splenic and jejunal infarction and focal nodular hyperplasia.

## Clinical presentation

A 31-year-old female presented with a 3-week history of abdominal pains associated with nausea, vomiting, loss of appetite and diarrhoea. She had no history of smoking or chronic illness or surgery. However, she had a 5-year history of using combined oral contraceptive pills (COCs). Rheumatoid factor, antinuclear body, protein C deficiency tests and platelet count were normal.

Endoscopy revealed gastric mucosa erythema and erosion and the gastric biopsy gave evidence of moderate chronic gastritis. Colonoscopy results showed features of ischaemic colitis but histological colonic biopsy report was normal. A left lower limb ultrasound Doppler scan was normal. The patient was sent for a triphasic CT of the abdomen.

## Scan protocol

The patient was scanned using a Somatom Definition AS, 128 slice, Siemens, Germany. For bowel preparation, Dulcolax tablets were given the night prior. Two hours before the scan, the patient was given 30 ml of Gastrografin dissolved in 1000 ml of water.

A 20-gauge cannula was inserted in the right median cubital vein. Hundred millilitres of Ultravist 370 contrast media was administered using a pump injector. A late arterial phase, portovenous phase and delayed phase scans were acquired 15 , 23 and 31 s after bolus tracking, respectively.

The late arterial phase was done to simultaneously evaluate the arterial vessels and to show the enhancement characteristics of the focal hepatic lesion. The portovenous phase was done to demonstrate enhancement of the focal liver lesion and organ perfusion characteristics. The delayed scans were done to show whether there was tumoral washout or if the focal hepatic lesion retained contrast in order to characterize it further. Multiplanar reconstructions of 3 mm (B20 smooth kernel, abdomen window) and 1 mm (B10 smooth kernel, abdomen window) for maximum intensity projections and 3D reconstructions were done.

## CT findings

**Figure 1.**
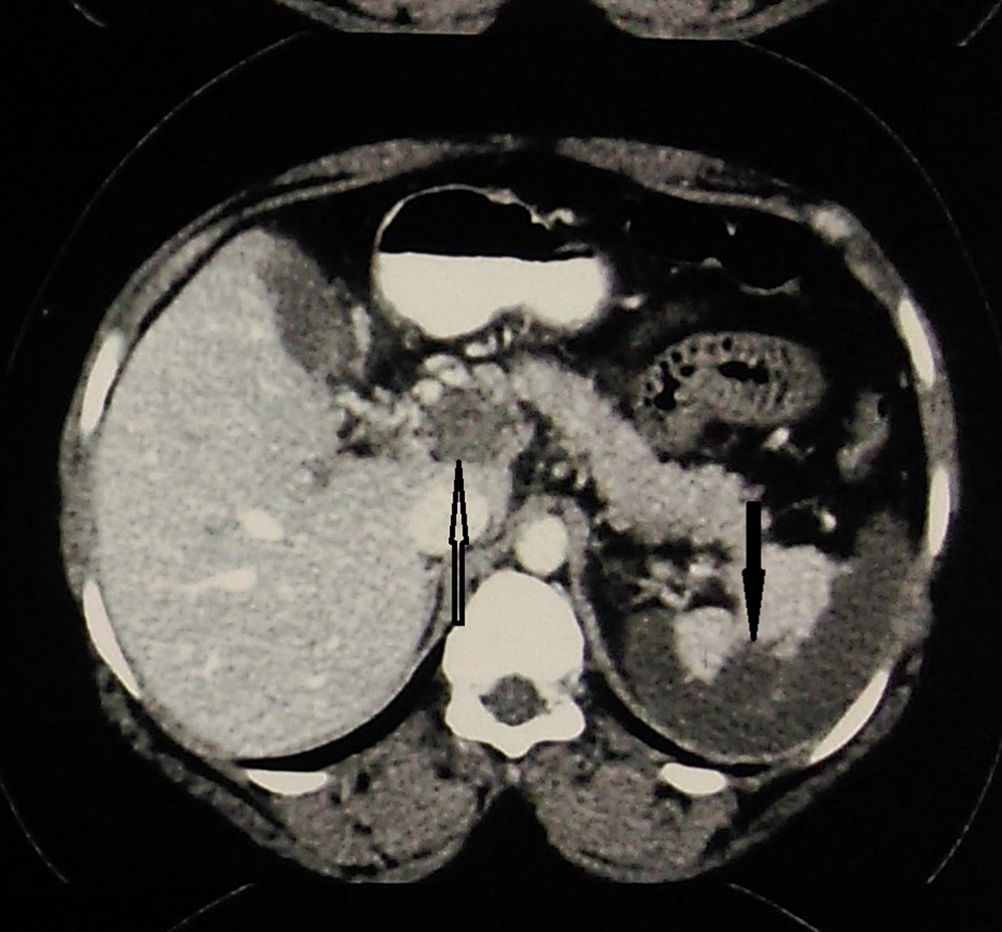
Axial reformatted venous phase image showing complete occlusive thrombosis of the portal vein (white arrow) and acute infarction of the spleen (dark arrow).

**Figure 2.**
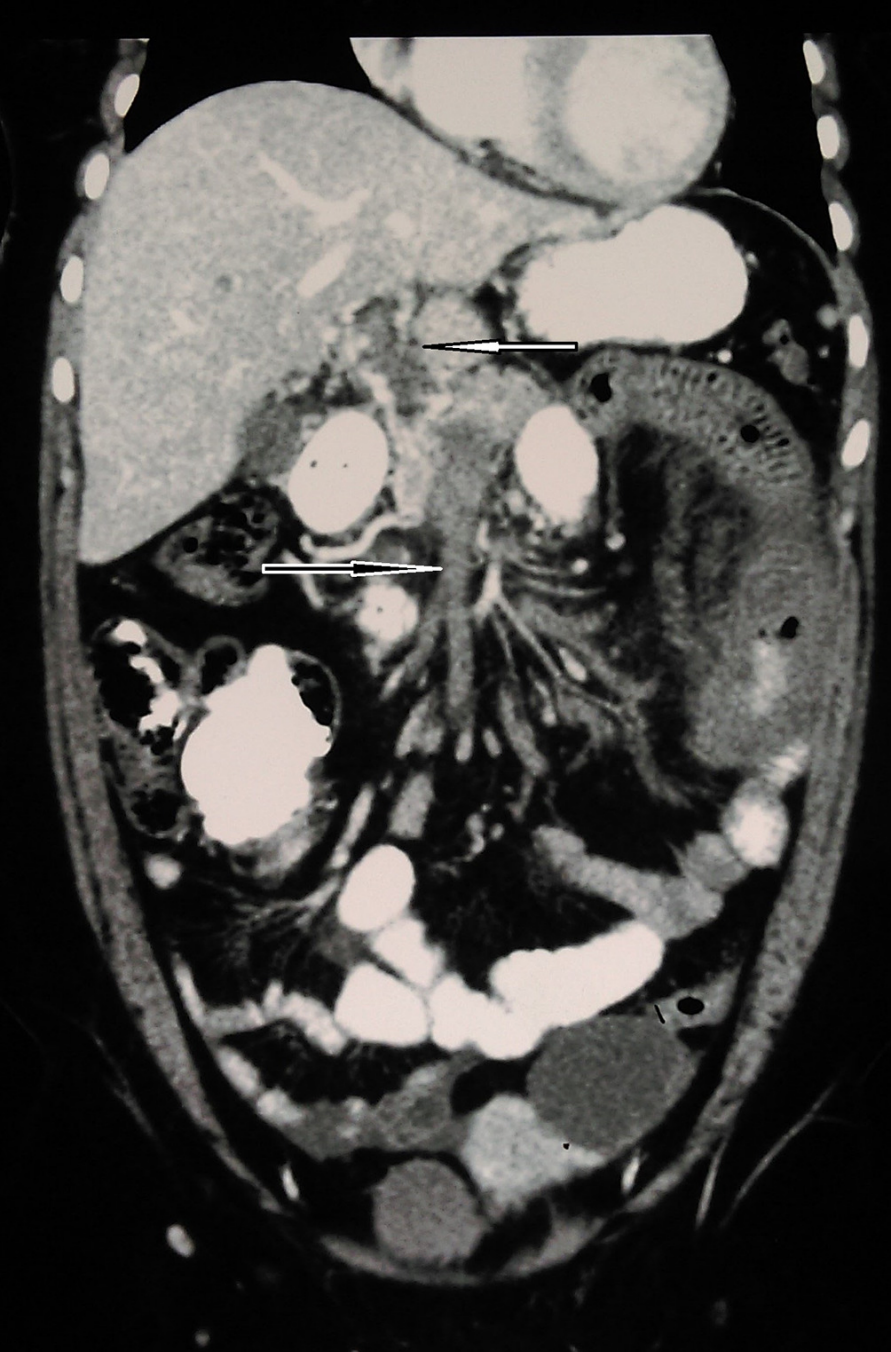
Coronal image reveals thrombotic occlusion of the superior mesenteric vein (dark arrow).

The CT abdomen showed complete occlusive thrombosis of the main portal vein (MPV) (Figure 1) and the superior mesenteric vein (SMV) (Figure 2). Enlarged collaterals running parallel to the MPV were also seen. The MPV had a diameter of 19 mm (normal < 12 mm). The SMV thrombosis extended into virtually all of its major large tributaries. The superior mesenteric artery and the inferior mesenteric artery were normal with good patency.

**Figure 3.**
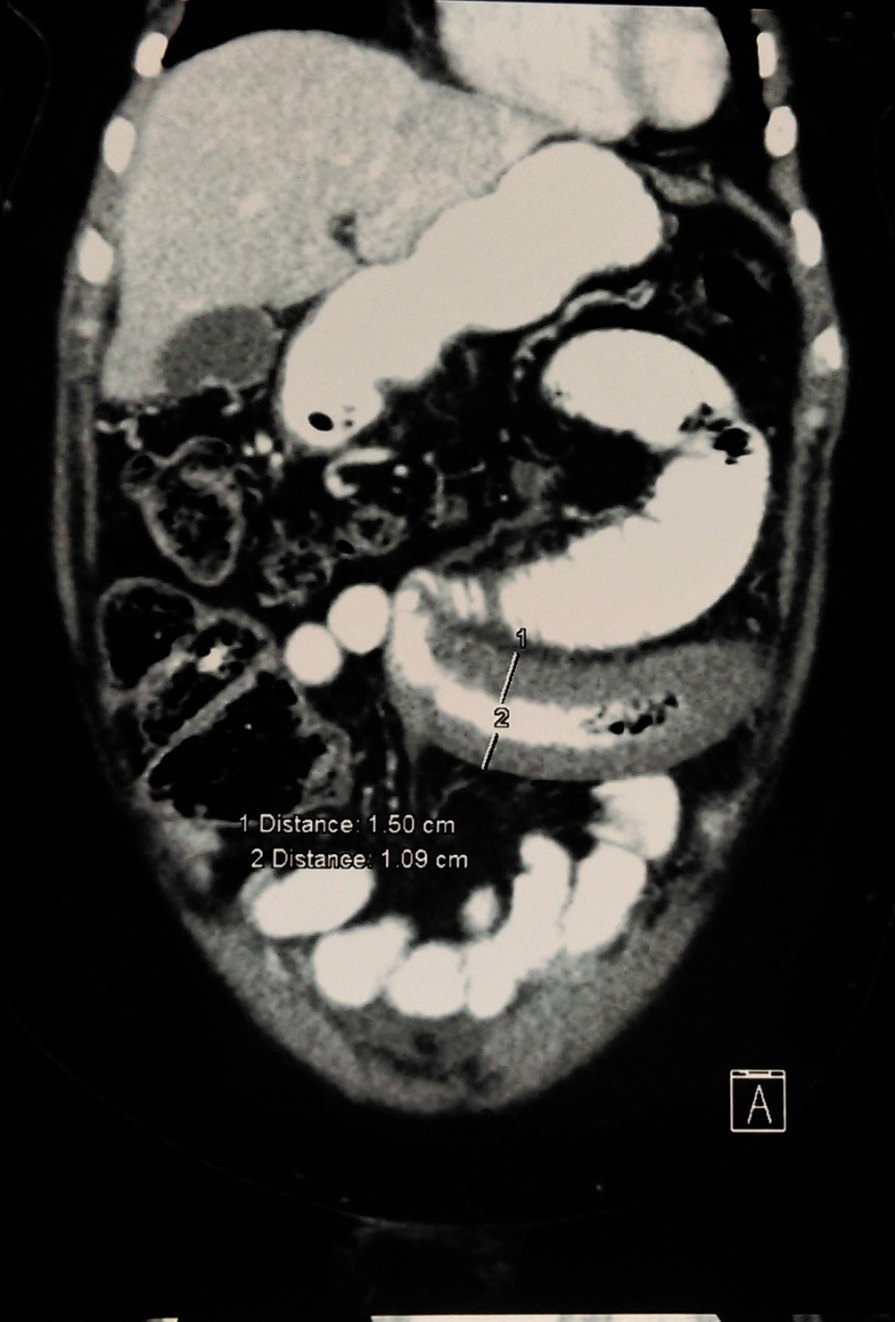
CT coronal reformatted image shows acute segmental jejunal infarction with smooth concentric non enhancing wall thickening.

There was an acute venous segmental jejunal infarction over a length of 20 cm. It had smooth concentric wall-thickening (16 mm) with reduced contrast enhancement (Figure 3). However, subtle enhancement was seen on delayed scans. Minimal oedema and trace ascites around this segment were also noted. No pneumatosis intestinalis or other features of bowel wall necrosis were seen. The rest of the small and large intestines were normal.

**Figure 4.**
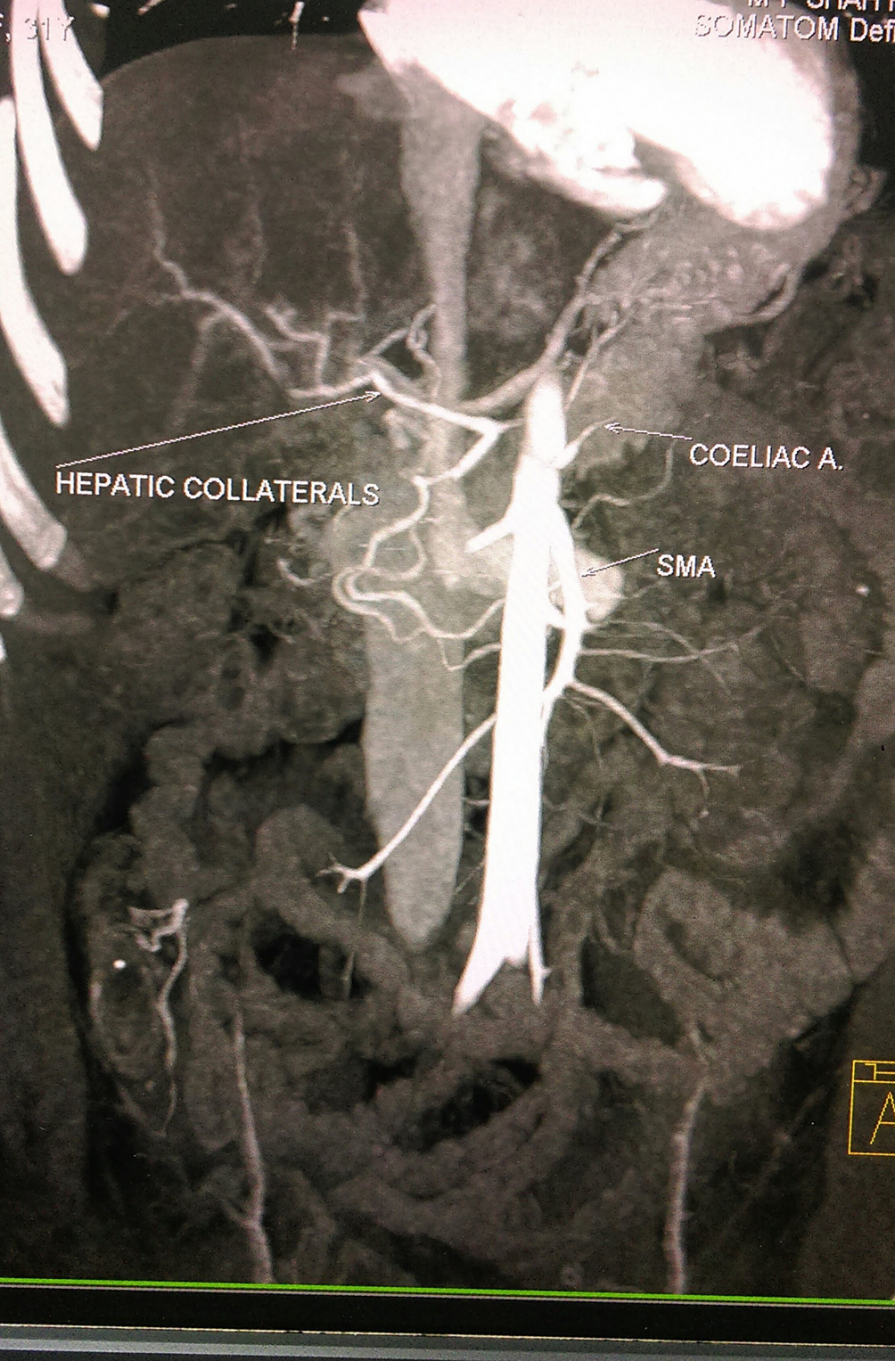
Maximum intensity projection image showing coeliac artery occlusion.

The coeliac trunk had small calibre with low attenuation filling defects in its lumen indicating partial thrombosis (Figure 4). Due to this ostial stenosis, there was diminished flow through this artery. The hepatic arteries showed some flow distally possibly from collaterals. The splenic artery was not visualized. This was associated with over 75% acute infarction of the spleen (Figure 1).

**Figure 5.**
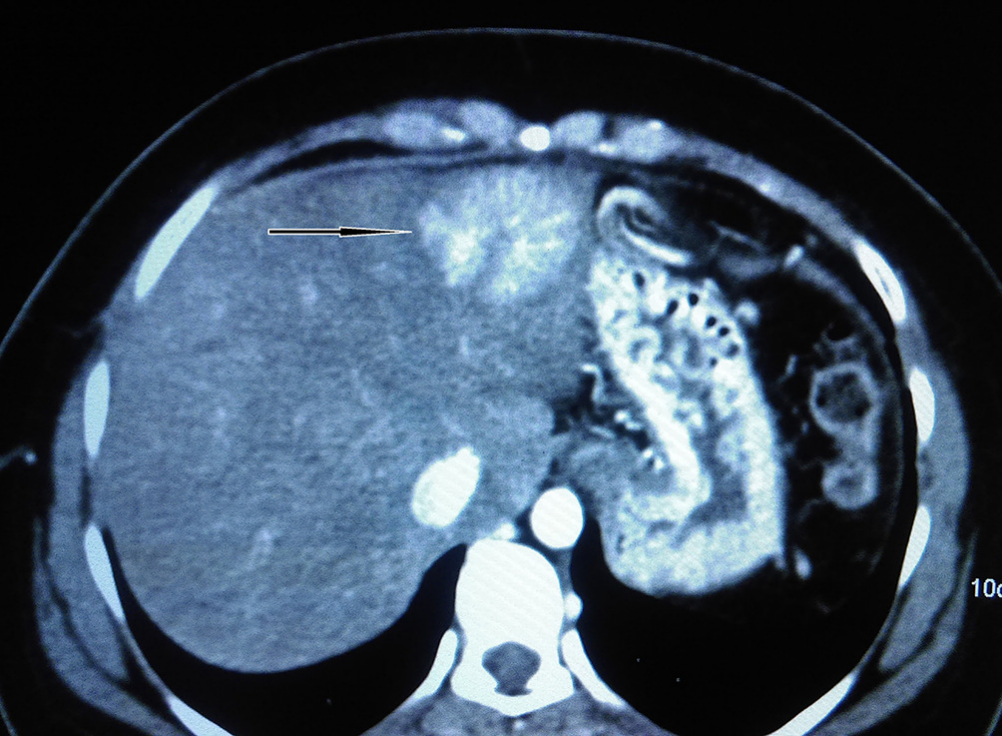
Axial reformatted image showing a segment II focal nodular hyperplasia lesion.

In the liver, a 4 × 5 × 4 cm segment II/IVA well-marginated lesion was demonstrated (Figure 5). It was hypervascular in the arterial phase and had radial septi. It washed out in the equilibrium phase and showed delayed enhancement of the septi. The CT characteristics of the lesion favoured a focal nodular hyperplasia (FNH). Fibrolamellar HCC was to be considered has a differential.

## Treatment

The patient was started on anticoagulant therapy (Xarelto 20 mg once daily for 3 months and Nexium 40 mg per oral twice daily). The patient was advised to stop COC use and consult a gynaecologist for further advice.

## Outcome/follow up

After 3 months of anticoagulant use, the patient was referred for a follow-up CT scan of the abdomen. On the follow-up CT scan, there was non-recanalization, atrophy and cavernous transformation of the portal vein (PV) and SMV. There was stricture formation in the previously ischaemic jejunal loop. Good compensatory hypertrophy of the non-infarcted splenic parenchyma was also noted. The segment II/IVA hepatic lesion remained the same.

The patient underwent surgery to resect the strictured jejunal loop after which she has succumbed due to complications of surgery a week later.

## Discussion

The untoward effects of COCs are thought to be related to both estrogen and progestin component. Progestins and estrogens are likely to potentiate arterial occlusion and venous occlusion, respectively. Thrombosis due to COCs can manifest in the arterial or venous circulation systems. The main adverse effects include thromboembolism, venous congestion, hepatic neoplasms such as hepatocellular adenomas and colitis.^1^

Other risk factors such as women with hereditary antithrombin III or protein C deﬁciency are more susceptible to develop thromboembolism due to COCs use. The presence of factor 5 Leiden mutations may make them susceptible to thromboembolic events.^2^

The mechanisms mediating the development of thromboembolism in COCs users are still equivocal. However, three mechanisms mediating the evolvement of thromboembolism have been postulated. COCs may trigger a hypercoagulable state through activation of the internal and external pathways of the coagulation cascade or by lowering of antithrombin III levels. COCs may cause antiﬁbrinolytic activity by reducing ﬁbrinolysis and inducing intimal hyperplasia.^1^

### Focal nodular hyperplasia

FNH is the second commonest benign hepatic lesion. In the recent past, FNH development was linked with the use of oral contraceptive use. This was because most reported incidences of FNH were higher in females than men but women also had a propensity of developing larger lesions.^3^

Recently, Mathieu et al^4^ conducted a research on the usage of oral contraceptive with relation to FNH. The study sample consisted of 216 women. The study results showed that neither the size of the lesion nor the number of FNH lesions was affected by COCs. The study also revealed that any alteration in the size of the lesions was rare and didn’t seem to depend on COC use.^4^

However, it should be noted that the 216 women enrolled for the study had been diagnosed with FNH lesions. The study didn’t provide an insight on the susceptibility of developing FNH lesions in women using COCs but with no diagnosis of FNH.

### Portal vein and superior mesenteric vein thrombosis

PV thrombosis refers to the complete or partial occlusion of blood flow in the PV. The etiological factors can be classified as local (70%) and systemic (30%) risk factors. Mesenteric venous thrombosis is classified as either primary or secondary. In primary mesenteric venous thrombosis no etiologic factor is found. The opposite is true for secondary mesenteric venous thrombosis.^5^

SMV thrombosis is due to primary hypercoagulable states which are either due to a drop in antithrombin III, protein C, or protein S or elevation of prothrombotic protein levels, such as activated protein C resistance (Factor V Leiden) or G20210A prothrombin gene mutation.^6^ Other causes include inheritable or acquired disorders of coagulation, cancer, inflammation, surgery, and cirrhosis and portal hypertension.^5^

Recent studies show that COCs cause 9 to 18% of mesenteric venous thrombosis in young women.^6^ This is because COCs have procoagulant, anticoagulant and fibrinolytic effects which potentiate thrombotic events.^6^

### Jejunal infarction

Due to portomesenteric thrombosis, there is associated jejunal or ileal segmental infarction (95% of the cases) and terminal ileum and right colon infarction (5% of the cases). Thrombosis affecting the inferior mesenteric vein is quite rare (less than 6% of the cases).^5^ Jejunal infarction arises from the venous thrombosis rather than arterial thrombosis^10^.

### Coeliac trunk thrombosis

Coeliac artery occlusion is a rare condition. However, coeliac artery occlusion or stenosis results to limited bowel ischaemia. The risk factors for coeliac artery occlusion are closely associated with the risk factors for thrombosis or embolism.^7^

Celiac trunk stenosis can be organic or functional. Organic-induced stenosis is usually due to atherosclerosis although infrequent causes are fibromuscular dysplasia, Buerger disease and arteritis nodosa.^8^ The commonest etiological factor attributable to functional-induced stenosis of the coeliac trunk is as result of compression by a median arcuate ligament.^8^

### Splenic infarction

Splenic infarction can be caused either by arterial or venous occlusion. The etiological factors are atrial fibrillation, ventricular aneurysm, sickle cell and heart valve diseases.^9^ Other causes are: acute pancreatitis, antiphospholipid syndrome, neoplasms, atherosclerosis, oral contraceptive and maladies related with hypercoagulability and surgery.^9^

Arul et al reported a case of splenic infarction secondary to coeliac artery trunk thrombotic occlusion due to combined oral contraceptive in a 14-year-old girl with no other risk factors of thromboembolism.^9^

## Conclusions

Complications associated with the use of COCs have well been documented in literature. However, most of these patients do not present with multiple complications of COCs use. Thus, this case report, to the best knowledge of the author, remains a rarity.

## Learning points

1. Multiple complications of combined oral contraceptive pills (COCs) use can simultaneously occur in a single patient.
2. Triphasic abdominal CT scan should be done to patients with a prior use of COCs to rule out hepatic lesions and thromboembolism.
3. Other causes of arterial and venous thrombosis should be excluded in patients with prior use of COCs.
4. Further studies should be done to provide an insight to the susceptibility of developing focal nodular hyperplasia or hepatic lesions in women using COCs but with no diagnosis of focal nodular hyperplasia or liver lesions.

Permission to write the case report was sought and granted by the institution through the head of department subject to the author ensuring the anonymity and confidentiality of the patients under study.

## Consent

Written informed consent for the case to be published (includes images, case history and data) was obtained from the patient(s) for publication of this case report, including accompanying images
